# Pasireotide-induced hyperglycemia in Cushing’s disease and Acromegaly: A clinical perspective and algorithms proposal

**DOI:** 10.3389/fendo.2024.1455465

**Published:** 2024-12-13

**Authors:** Przemysław Witek, Marek Bolanowski, Adam Krętowski, Aleksandra Głowińska

**Affiliations:** ^1^ Department of Internal Medicine, Endocrinology, and Diabetes, Medical University of Warsaw, Warsaw, Poland; ^2^ Department of Endocrinology and Internal Medicine, Medical University of Wroclaw, Wroclaw, Poland; ^3^ Department of Endocrinology, Diabetology, and Internal Medicine, Medical University of Bialystok, Bialystok, Poland; ^4^ Recordati Rare Diseases, Central and Eastern Europe, Warsaw, Poland

**Keywords:** Acromegaly, antidiabetic therapy, Cushing’s disease, GLP-1 receptor agonists, Hyperglycemia, pasireotide, SGLT-2 inhibitors

## Abstract

Pasireotide is an effective treatment for both Cushing’s disease (CD) and acromegaly due to its ability to suppress adrenocorticotropic hormone and growth hormone, and to normalize insulin-like growth factor-1 levels, resulting in tumor shrinkage. However, it may also cause hyperglycemia as a side effect in some patients. The aim of this study was to review previous recommendations regarding the management of pasireotide-induced hyperglycemia in patients with CD and acromegaly and to propose efficient monitoring and treatment algorithms based on recent evidence and current guidelines for type 2 diabetes treatment. In about 25% of patients with CD and 50% of patients with acromegaly, pasireotide-induced hyperglycemia does not require drug therapy or can be managed with diet and oral antidiabetic agents. The risk of pasireotide-induced hyperglycemia is higher in patients with diabetes or prediabetes at baseline. Moreover, pasireotide used in the treatment of CD may lead to more frequent and difficult-to-treat glycemic disorders than those observed in acromegaly. Based on the pathomechanism of hyperglycemia, we suggest using metformin as the first-line therapy, followed by glucagon-like peptide-1 and/or sodium-glucose co-transporter-2 inhibitor, and finally insulin in patients with pasireotide-induced hyperglycemia. We propose algorithms for the management of glucose metabolic disorders caused by pasireotide treatment in patients with CD and acromegaly, including those with chronic kidney disease and at high cardiovascular risk.

## 1 Introduction

Cushing’s disease (CD) and acromegaly are rare pituitary disorders that significantly affect mortality risk and quality of life of patients (1, 2). The reported prevalence of CD ranges from 12 to 62 cases per million, and that of acromegaly – from 28 to 137 cases per million (3, 4). Both diseases are typically caused by benign pituitary neuroendocrine tumors (adenomas) that overproduce hormones. CD is characterized by chronic hypercortisolemia secondary to dysregulated adrenocorticotropic hormone (ACTH) release. In contrast, acromegaly is characterized by the increased secretion of growth hormone (GH) and, consequently, insulin-like growth factor-1 (IGF-1), which serves as a peripheral mediator of GH action (5, 6). In the absence of treatment, prolonged endocrine abnormalities lead to various cardiovascular and metabolic complications as well as comorbidities (1, 2).

The typical clinical symptoms of CD include central obesity, “moon face”, excess fat deposition in the back of the neck, hirsutism, muscle weakness, and thin skin characterized by easy bruising. CD is associated with hypertension, impaired glucose tolerance or type 2 diabetes mellitus (T2DM), osteoporosis, frequent infections, and psychiatric complications (7, 8). Despite increased cardiometabolic risk in patients with CD even after the biochemical remission of hypercortisolism, the median survival is significantly prolonged after cure (4, 9, 10). The clinical symptoms of acromegaly include changes in appearance, hand and foot enlargement, postural changes, and spine deformities. The consequences of acromegaly include arterial hypertension, cardiac hypertrophy, secondary hormonal dysfunctions, osteoporosis, and metabolic disorders (such as prediabetes, T2DM, and lipid disorders), mental changes, and a worsening of the quality of life (6, 11). Active acromegaly shortens survival by 10 years (12). The major causes of premature mortality are cardiovascular diseases, respiratory system disorders, and neoplasms (6, 13). However, evidence shows that mortality rates associated with acromegaly have been significantly reduced due to the availability of effective therapeutic options that provide biochemical control (14, 15).

As glucose metabolism is impaired in both CD and acromegaly, T2DM is a common complication of both diseases and has been classified by the American Diabetes Association (ADA) as a “specific type of diabetes due to other causes” (16–18). It is estimated that approximately half of patients with CD and acromegaly have diabetes and about 30% of them have prediabetes (16, 19). The prevalence of T2DM in patients with CD is probably underestimated because an oral glucose tolerance test for the identification of glucose metabolic disorders is not performed in all patients (19). The activation of key gluconeogenic enzymes by elevated cortisol levels in CD results in increased glucose synthesis from noncarbohydrate sources, particularly proteins. Chronic exposure to cortisol in patients with CD may affect pancreatic beta cell function, glucose uptake and storage in muscles and fat, gluconeogenesis and glycogenolysis in the liver, and lipid metabolism in adipose tissue, leading to insulin resistance (2, 19, 20). Cortisol excess also affects GH and IGF-1 levels, resulting in an increase in visceral fat and insulin resistance (19). In acromegaly, excess GH promotes insulin resistance in the liver and peripheral tissues, counteracting the inhibitory effect of insulin on gluconeogenesis and enhancing endogenous glucose production (21, 22). Moreover, GH stimulates lipolysis in the adipose tissue, increasing free fatty acid concentrations and decreasing glucose utilization in skeletal muscles (16, 21, 22). In addition to the diabetogenic effect of GH outweighing the insulin-sensitizing effect of IGF-1, GH and IGF-1 act synergistically to increase insulin secretion, resulting in hyperinsulinemia (16). The cardiovascular risk in CD and acromegaly depends on diabetes control and the presence of diabetic complications (23, 24).

In patients with CD and acromegaly, the therapy of choice is selective transsphenoidal adenomectomy, which can result in a rapid, long-lasting remission in over 60% of cases (25, 26). In cases where neurosurgery fails or the tumor recurs, medical therapy is necessary. Successful neurosurgery was shown to improve glucose tolerance and normalize glucose metabolism in approximately half of patients with CD and acromegaly with concomitant diabetes (27, 28). The effect of medical therapies on glucose metabolism depends on the type of the medication administered (2, 19, 21, 29). Most drugs used in the treatment of CD were reported to improve glucose metabolism (osilodrostat, metyrapone, ketoconazole, mitotane, etomidate, and mifepristone) (2, 19). First-generation somatostatin analogs, octreotide and lanreotide, have a minimal clinical impact on glucose metabolism in patients with acromegaly (29–31). Other medications used in the treatment of acromegaly, such as GH receptor antagonists (pegvisomant) or dopamine agonists (cabergoline, bromocriptine), have some beneficial effects on glycemia (21, 32). Recently, it was found that pegvisomant may mitigate the worsening of glucose metabolism caused by long-acting pasireotide in some patients (33). Pasireotide, a second-generation multi-receptor-targeted somatostatin analog, which was approved for use both in patients with CD and acromegaly, suppresses not only the secretion of ACTH and GH but also of glucagon-like peptide-1 (GLP-1), glucose-dependent insulinotropic polypeptide (GIP), and insulin, along with the inhibition of the incretin effect (2, 19, 21, 29). In some patients with CD and acromegaly, the resulting imbalance between insulin and glucagon may lead to pasireotide-induced hyperglycemia (34, 35).

The aim of this study was to review current recommendations for the management of pasireotide-induced hyperglycemia in patients with CD and acromegaly and to propose algorithms for efficient monitoring and treatment of these patients based on recent evidence.

## 2 Pasireotide-induced hyperglycemia

Pasireotide was introduced to the market 20 years ago and approved for use in patients with CD and acromegaly who are ineligible for surgery or have persistent or recurrent disease after initial surgery (34, 36, 37). Real-world data showed that pasireotide applied monthly as a long-acting intramuscular formulation triggered a biochemical response in about 40% of patients with CD and in 54% to 59% of patients with acromegaly after 6 months or longer of treatment (38–41). Pasireotide inhibits the secretion of ACTH and GH due to its binding to somatostatin receptor (SSTR) subtypes expressed by corticotroph and somatotroph pituitary tumors (34, 42). In contrast to first-generation somatostatin analogs, pasireotide exhibits high affinity for all SSTR subtypes, except SSTR4. Its affinity for SSTR5 is higher than that for SSTR2 (34, 43, 44). Due to its broad binding profile, pasireotide can be used for CD treatment and was shown to have greater clinical efficacy in acromegaly than octreotide (45, 46). However, the binding profile of pasireotide is associated with a relatively high incidence of hyperglycemia (34, 47). Since the binding affinity of pasireotide to SSTR5 present in pancreatic beta cells is higher than to SSTR2 predominant in alpha cells, the drug reduces insulin secretion but has minimal effect on glucagon secretion, with no change in insulin resistance (34, 40, 42, 47). Additionally, pasireotide suppresses intestinal incretin release, leading to poor glycemic control (42, 47). In uncontrolled CD or acromegaly, reduced insulin levels associated with pasireotide cannot counterbalance the impaired insulin sensitivity (42). Following pasireotide discontinuation, glycemia returns to the pretreatment levels (48, 49). The effect of pasireotide on the lipid profile and adipose tissue dysfunction as factors increasing cardiometabolic risk has not yet been fully elucidated (50).

The frequency of hyperglycemia-related adverse events induced by pasireotide in patients with CD seems to be higher than in those with acromegaly (68.4–73.0% vs 57.3–67.0%) (34, 42, 51). However, data from clinical trials are inconsistent regarding the prevalence of hyperglycemia and diabetes in patients with CD and acromegaly treated with pasireotide (**Table 1**). Clinical practice shows that pasireotide used in the treatment of CD may lead to more difficult-to-treat glucose metabolic disorders than those occurring in acromegaly (52). The rate of study discontinuation associated with hyperglycemia was higher in the CD trials (5.3%-6%) than in the acromegaly trials (3.4%-3.8%) (42). It should be noted that in the trials compared, patients with CD received subcutaneous pasireotide, while patients with acromegaly received long-acting pasireotide, and this may have affected the results (42).

**Table 1:** The prevalence of hyperglycemia and diabetes in patients with Cushing’s disease and acromegaly during treatment with pasireotide.

Disease	Prevalence during treatment with pasireotide (%)		Total number of patients (N)	Type of study	Reference
	Hyperglycemia	Diabetes			
Cushing’s disease	48	21	150	Phase III clinical trial	(38)
	40	18	162	Phase III clinical trial	(46)
	42.8	42.8	14	Phase III clinical trial (single center)	(63)
Acromegaly	6.7–10	5–16.7	30	Phase II clinical study and extension	(89, 90)
	32	23	125	Phase III PAOLA clinical trial	(62)
	39.7-40.3	31.7- 40.3	125	Extension to a phase III PAOLA clinical study	(91)
	28.7	19.1	178	Prospective multicenter study	(45)
	22.7	13.6	44	Open-label, multicenter, safety monitoring program	(92)
	46.2	46.2	39	Two-center retrospective study	(39)
	52.9	32.4	34	Observational, retrospective multicenter study	(33)

The risk of pasireotide-associated hyperglycemia is increased in patients classified as diabetic or prediabetic at baseline (37, 41). The adverse effect of pasireotide on glucose metabolism is typically observed within the first 3 months and stabilizes during long-term treatment, likely due to improvement in insulin sensitivity (2, 19, 53, 54). In approximately 25% of patients with CD and 50% of patients with acromegaly, drug therapy is not required or patients can be relatively easily managed with diet and oral antidiabetic agents (34, 52, 55). According to current guidelines, patients with CD or acromegaly who start or discontinue treatment with pasireotide, or require dose adjustment, should be adequately managed and regularly monitored for blood glucose levels (42).

The current therapeutic management of pasireotide-induced hyperglycemia is based on expert opinions and general recommendations for typical T2DM. Previously proposed algorithms for the monitoring and treatment of pasireotide-induced hyperglycemia in patients with CD and acromegaly were described in relation to our own proposal in the Discussion section.

## 3 Predictors of pasireotide-induced hyperglycemia

The baseline glycemic status of patients with CD and acromegaly predisposes to the development of T2DM before the initiation of pasireotide treatment and could be predictive of the severity of hyperglycemia associated with treatment (42). However, not all patients develop pasireotide-induced hyperglycemia. In CD patients, high glucagon levels, glycated hemoglobin (HbA1c) levels higher than 34.5 mmol/mol (> 5.3%), and a glucose peak after pasireotide administration higher than 9 mmol/L (> 162 mg/dL) were associated with a higher risk of T2DM during pasireotide treatment. There were no differences in age, fasting plasma glucose (FPG), or disease severity between patients with and without T2DM (56). Similarly, in a recent trial, increased baseline levels of HbA1c, but not FPG, were predictive of hyperglycemia in patients with CD (51). However, in other studies, baseline FPG levels higher than 100 mg/dL predicted T2DM in pasireotide-treated patients, either with CD or acromegaly (57, 58).

Glucose metabolic disorders during pasireotide treatment were less common in acromegaly patients with a younger age (<40 years), normal glucose tolerance, and no history of hypertension or dyslipidemia (59). Additionally, age, high HbA1c levels, and the presence of prediabetes or diabetes were identified as risk factors for pasireotide-induced hyperglycemia in patients with acromegaly in a *post hoc* analysis of the phase IV B2219 study (57). Gadelha et al. also identified IGF-1 levels as a predictor of diabetes in acromegaly during pasireotide treatment (41). Moreover, it was reported that increased pasireotide dose and a body mass index of 25 kg/m^2^ or higher were associated with a higher risk of pasireotide-induced hyperglycemia in patients with acromegaly (42, 60). A phase IV trial recently demonstrated that elevated HbA1c and FPG levels at baseline, as well as a history of diabetes or prediabetes, predicted the need for antihyperglycemic treatment in patients with acromegaly (51, 52). Due to the ease of testing, HbA1c is suggested as the best predictor of pasireotide-induced hyperglycemia (56).

## 4 Management of pasireotide-induced hyperglycemia in clinical practice

Before starting pasireotide treatment, a detailed analysis of glucose metabolism should be performed, including the assessment of FPG and HbA1c levels (42). Self-monitoring of blood glucose, control of dietary habits, and appropriate physical activity are recommended in all patients (61). Blood glucose levels should be monitored weekly for the first 2 to 3 months after starting pasireotide and for the first 2 to 6 weeks after dose escalation (42). In patients with diabetes (long-standing or newly diagnosed), it is necessary to initiate or optimize antidiabetic therapy. Failure of glucose metabolic control during pasireotide treatment requires modification of antidiabetic therapy (62). As few studies have addressed pasireotide-induced hyperglycemia to date, the optimal approach to its management is still being sought (63).

In the phase IV B2219 study, patients with CD and acromegaly treated with pasireotide received metformin as the first-line therapy. Patients with increased FPG were randomized to incretin-based therapy (sitagliptin – dipeptidyl peptidase-4 [DPP-4] inhibitor followed by liraglutide – GLP-1 receptor agonist [GLP-1 RA]) or insulin for 16 weeks. Treatment with insulin was instituted as rescue therapy to achieve optimal glucose control. The primary objective was to evaluate the difference in change in HbA1c between the incretin and insulin arms. The study demonstrated the advantage of incretin therapy over insulin therapy (52). Incretin-based therapy was also associated with fewer episodes of hypoglycemia and cholelithiasis than insulin therapy (52). Metformin followed by incretin-based therapy was the effective treatment for pasireotide-induced hyperglycemia. However, the study had some limitations regarding patients with CD. The group was not large enough to perform separate analyses for hypercortisolemia (59 patients with CD vs 190 patients with acromegaly). Therefore, some conclusions are based on joint analyses of the group with CD and acromegaly, although these are two different diseases (50). In addition, different formulations of pasireotide were used for CD (subcutaneous pasireotide) and acromegaly (long-acting pasireotide) (50). The dose for CD was 2x600 μg subcutaneously, which could be increased to 2x900 μg subcutaneously (50). Patients with CD after pasireotide initiation were more likely to receive insulin as rescue therapy than patients with acromegaly (50). Moreover, patients with CD who were randomized to the incretin or insulin arm had higher FPG and HbA1c levels than those with acromegaly (50).

In a study of healthy volunteers, vildagliptin (DPP-4 inhibitor) and liraglutide (GLP-1 RA) were the most effective agents in minimizing pasireotide-associated hyperglycemia (64). This suggests that the incretin-based therapy may correct the impairment of insulin-to-glucagon ratio caused by pasireotide treatment. Both DPP-4 inhibitors and GLP-1 RAs reduce glucagon levels in a glucose-dependent manner (65). In addition, GLP-1 RAs were shown to be effective in controlling symptoms of persistent hypercortisolemia in patients with CD, such as increased appetite, obesity, and dyslipidemia (65). The role of long-acting GLP-1 RAs has not been well established, but based on their high efficacy and safety in T2DM, it is expected that they may be equally effective in pasireotide-induced hyperglycemia in patients with CD and acromegaly (66). Long-acting pasireotide administered once a month in combination with GLP-1 RA (e.g., semaglutide or dulaglutide) administered once a week may improve treatment outcomes and patient comfort (34). Moreover, the mechanism of action of tirzepatide, a dual GIP and GLP-1 RA recently approved for use in patients with T2DM, indicates its high potential in the treatment of pasireotide-associated hyperglycemia. Despite limited evidence, it can be hypothesized that it will become a therapeutic option in patients with CD or acromegaly (67, 68).

Although there is still a lack of sufficient data addressing the use of sodium-glucose cotransporter-2 (SGLT-2) inhibitors in patients treated with pasireotide, the results of SGLT-2 inhibitor administration in patients with diabetes and acromegaly have already been reported (69). According to Zaina et al., treatment with SGLT-2 inhibitors could be considered for T2DM in patients with acromegaly in the following cases: in patients with previously diagnosed T2DM and controlled acromegaly after surgery, in patients with T2DM and controlled acromegaly treated with long-acting somatostatin analogs or pegvisomant, and patients treated with long-acting pasireotide with worsening hyperglycemia or new-onset diabetes. In the case of patients with uncontrolled GH/IGF-1 levels or severe hypercortisolism, caution and careful monitoring is needed when using SGLT-2 inhibitors due to the increased risk of diabetic ketoacidosis and genitourinary fungal infections (69, 70). SGLT-2 inhibitors can be used in pasireotide-treated patients with CD or acromegaly with worsening hyperglycemia or newly diagnosed diabetes, with metformin as monotherapy or in combination with DPP-4 inhibitors or GLP-1 RAs (69, 71). SGLT-2 inhibitors exert antidiabetic effects via glycosuria and improvement of insulin sensitivity in skeletal muscles and adipose tissue. Moreover, SGLT-2 inhibitors reduce cardiovascular mortality and heart failure exacerbations (71, 72). Therefore, clinical guidelines recommend the use of these drugs for patients with T2DM and cardiovascular diseases.

In the phase III C2402 (PAOLA) trial, patients with acromegaly received metformin, a sulfonylurea derivative (glimepiride), and insulin (62). Hyperglycemic control defined by the ADA and the European Association for the Study of Diabetes (EASD) as HbA1c < 7% (53 mmol/mol) was achieved in 73% of patients receiving long-acting pasireotide at a dose of 40 mg and in 60% of patients receiving long-acting pasireotide at a dose of 60 mg (59, 73, 74). However, combination therapy of metformin with sulfonylureas and/or pioglitazone is not recommended for pasireotide-induced hyperglycemia. In the case of sulfonylureas, their mechanism of action is associated with a high risk of hypoglycemia, while in the case of pioglitazone, the risks include weight gain, fluid retention, and bone fractures. All of these adverse effects are particularly unfavorable in patients with CD (29, 61). Moreover, despite the multiple beneficial cardiovascular effects of pioglitazone, its use is not recommended in patients with heart failure because it causes renal sodium retention and vasodilation, resulting in fluid retention (70, 75, 76). Due to impaired pancreatic insulin secretion and incretin response during pasireotide treatment, GLP-1 RAs and SGLT-2 inhibitors appear to be better therapeutic options.

## 5 Monitoring and treatment algorithms

### 5.1 Patients with CD

**Figure 1:**
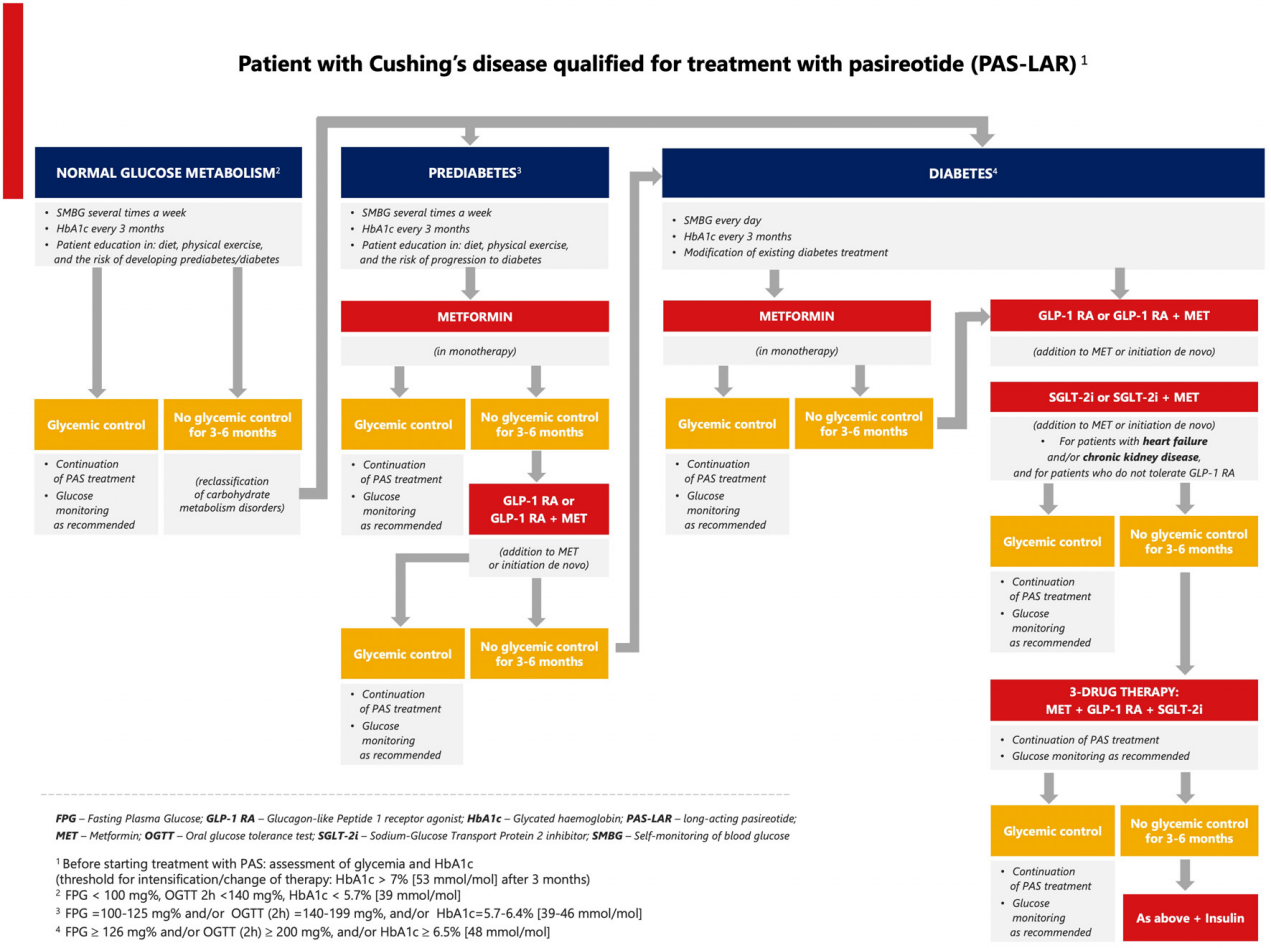
Proposed algorithm for the management of pasireotide-induced hyperglycemia in Cushing’s disease.

Every patient with CD should be prepared for pasireotide treatment in terms of proper education, blood glucose monitoring, lifestyle modification, and the escalation of oral antidiabetic therapy. Based on the available data from clinical studies and current clinical practice in T2DM, we suggest that metformin should be the first-line therapy for patients treated with pasireotide with prediabetes, followed by a GLP-1 RA. Metformin combined with a GLP-1 RA should be considered for patients with CD and T2DM, especially for those at high cardiovascular risk (**Figure 1**) (52). SGLT-2 inhibitors are recommended for patients with chronic kidney disease and the predominant pattern of heart failure as well as for patients who do not tolerate GLP-1 RAs. If glycemic control is not achieved, triple therapy should be used (metformin, GLP-1 RA, and SGLT-2 inhibitor). Clinical data demonstrated that metformin can be used successfully in the initial treatment of pasireotide-induced hyperglycemia in patients with CD (52, 61). It improves insulin sensitivity and lipid profile, reduces body weight, has a good safety profile, and does not interact with pasireotide. The combined therapy with metformin and a GLP-1 RA is recommended in patients with cardiac burden and cardiovascular risk factors based on the results of the B2219 study and the cardiovascular outcomes trials of antidiabetic drugs (52, 77). GLP-1 RAs are effective by supplementing the peptide that is missing due to the inhibitory effect of pasireotide on GLP-1 secretion. In contrast to GLP-1-based therapy, the effect of a DPP-4 inhibitor is hampered by pasireotide because a DPP-4 inhibitor can only potentiate the effect of endogenous GLP-1, whose concentration is significantly reduced during pasireotide treatment. Based on the results of the B2219 study, a DPP-4 inhibitor can be used as a supportive drug for T2DM in hypercortisolemia but without a strong effect on pasireotide-induced hyperglycemia (52). In patients with CD and acromegaly, a trend toward better metabolic control was observed in the incretin arm compared with the insulin arm, with a lower frequency of hypoglycemic episodes (52). Metformin, used in combination with other oral drugs (sulfonylurea or pioglitazone), might be an alternative to modern pharmacotherapy in the absence of financial resources.

### 5.2 Patients with acromegaly

**Figure 2:**
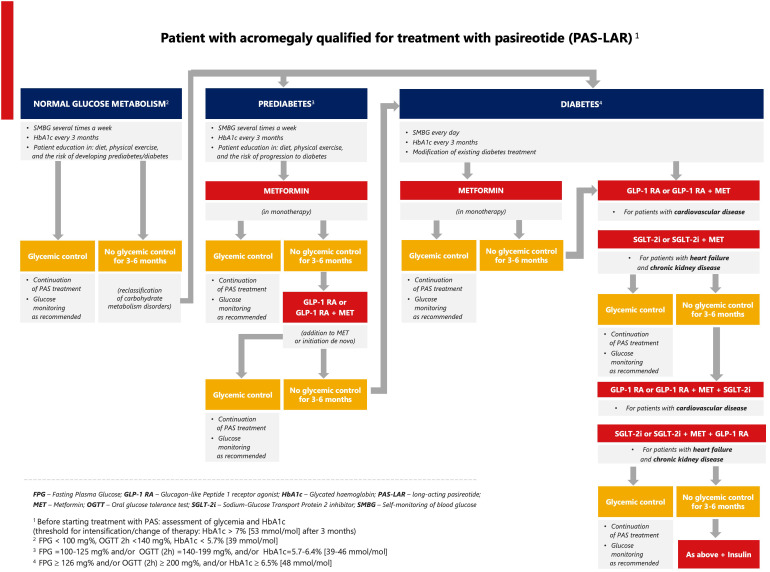
Proposed algorithm for the management of pasireotide-induced hyperglycemia in acromegaly.

Based on the limited clinical data (51, 52, 78) and the ADA/EASD recommendations (77, 79–81), we propose an algorithm for pasireotide-induced hyperglycemia in patients with acromegaly in relation to cardiovascular and renal disease (**Figure 2**). First, we propose individualized self-monitoring of glycemia at different time intervals according to glucose metabolic status (normal, prediabetes, diabetes). For patients with normal blood glucose levels or prediabetes, dietary counseling along with physical activity is recommended. In the case of prediabetes, metformin should be used first in monotherapy, and if it is ineffective, then in combination with a GLP-1 RA. In patients diagnosed with diabetes, antidiabetic therapy should be modified or intensified. If glycemic thresholds [FPG > 126 mg/dL and/or postprandial glucose > 200 mg/dL, and/or HbA1c > 7% (>53 mmol/mol)] are exceeded, an SGLT-2 inhibitor is suggested in patients with heart failure or kidney disease, and a GLP-1 RA in patients with cardiovascular disease. In both cases, metformin can be added. The next steps include combinations of the above therapies. Another therapeutic option is insulin as monotherapy or combination therapy. The addition of a sulfonylurea or pioglitazone is acceptable in case of lack of financial resources for modern therapy.

## 6 Discussion

Considering underlying physiology seems to be paramount when initiating antidiabetic treatment in patients with CD and acromegaly (80). Various algorithms have been proposed for the management of pasireotide-induced hyperglycemia. Ceccato et al. suggested metformin as the first-line therapy, followed by a DPP-4 inhibitor, GLP-1 RA, and insulin (82). Although this algorithm was originally intended for the treatment of CD, it has been commonly applied to patients with acromegaly, but with short-acting pasireotide (82). According to Colao et al., pasireotide-induced hyperglycemia in patients with CD should be treated first with metformin alone and then in combination with a DPP-4 inhibitor, with a switch to a GLP-1 RA and initiation of insulin, as needed (61). A similar algorithm was previously described by Reznik et al. based on literature data (83).

In patients with acromegaly, Coopmans et al. suggested a DPP-4 inhibitor first, followed by metformin in mild diabetes. In the absence of a DPP-4 inhibitor, metformin should also be used. In the second step, GLP-1 RA should be used, or a sulfonylurea if GLP-1 RA is unavailable, and finally, insulin (84). Recently, Zaina et al. recommended treatment with an SGLT-2 inhibitor or a GLP-1 RA after metformin in patients after surgery or in those with pharmacologically controlled acromegaly (72). For patients with poorly controlled acromegaly, an SGLT-2 inhibitor was not recommended due to an increased risk of diabetic ketoacidosis (72). The most recent expert consensus on the management of pasireotide-induced hyperglycemia in patients with acromegaly recommended incretin-based drugs (DPP-4 inhibitors in patients at low cardiovascular risk and GLP-1 RAs in patients at high cardiovascular risk) as an alternative to first-line monotherapy with metformin, emphasizing the need for early dual therapy with proven effects on cardiovascular and renal outcomes (78). DPP-4 inhibitors were also proposed as an alternative to GLP-1 RAs in the setting of gastrointestinal side effects (78). However, this expert opinion is still not recognized as the guideline for the treatment of pasireotide-induced hyperglycaemia. As DPP-4 inhibitors appear to be ineffective in pasireotide-induced hyperglycemia due to their mechanism of action, we decided not to include them in our algorithms.

In the approach proposed by Guarnotta et al., combined therapy with metformin and GLP-1 RA should be used as a first step in pasireotide-induced hyperglycemia in acromegaly and CD due to cardiovascular and weight loss benefits (70). This is generally consistent with our proposal for patients with diabetes. However, in contrast to our approach, Guarnotta et al. did not recommend the use of SGLT-2 inhibitors due to the risk of genitourinary mycotic infections in patients with severe hypercortisolism and the risk of ketoacidosis in patients with acromegaly (70, 72). Nevertheless, this risk does not seem to be high and it is known that infections and ketoacidosis may be exacerbations due to inadequate management of T2DM as well, requiring separate and appropriate treatment. Moreover, Guarnotta et al. included DPP-4 inhibitors in the algorithms as an alternative in case of GLP-1 RA intolerance (70). According to us, DPP-4 inhibitors are not recommended as first-line therapy because they only increase the concentration of endogenous GLP-1, which is considerably reduced during pasireotide treatment.

Pasireotide-induced hyperglycemia in patients with CD and acromegaly has been classified by the ADA as a “specific type of diabetes due to other causes” (18). The therapeutic management of T2DM in CD or acromegaly is similar to that of T2DM in patients with cardiovascular risk. According to the current Polish recommendations for diabetes care, GLP-1 RAs and/or SGLT-2 inhibitors with or without metformin should be considered as first-line treatment (85). The cardiovascular and renal protective effects of SGLT-2 inhibitors or GLP-1 RAs should be considered especially in patients with atherosclerotic cardiovascular disease, heart failure, or chronic kidney disease (85). Metformin is the first-line treatment for hyperglycemia in patients at low cardiovascular risk. If hyperglycemia is exacerbated by pasireotide treatment and there is increased cardiovascular risk, GLP-1 RAs (alone or in combination with metformin) should be considered as first-line therapy (77, 79, 86). SGLT-2 inhibitors should be used particularly in patients with chronic kidney disease and heart failure (85). The EMPA-REG OUTCOME trial in patients with T2DM at high cardiovascular risk showed that the addition of empagliflozin to standard therapy reduced the risk of cardiovascular death, non-fatal myocardial infarction, or non-fatal stroke by 14% and all-cause mortality by 32% (87). Similarly, diabetic patients treated with canagliflozin had a lower risk of cardiovascular events (88). In addition to comorbidities, obesity, and the risk of hypoglycemia, clinicians should also consider the patient’s financial capabilities as a factor in the selection of an antihyperglycemic drug, as not all modern agents are reimbursed (85).

The limitation of our algorithm proposals is the lack of real-world data and well-designed clinical research investigating the efficacy and safety of antidiabetic medications in patients with CD and acromegaly, mainly due to the fact that the patient populations are small for these rare diseases. In addition, not all patients with CD or acromegaly are treated with pasireotide and not all patients treated with pasireotide develop hyperglycemia. The proposed algorithms for pasireotide-induced hyperglycemia treatment are based mainly on current recommendations for T2DM therapy, a few clinical studies available for CD and acromegaly, and clinical practice.

## 7 Conclusions

The management of pasireotide-induced hyperglycemia in patients with CD or acromegaly should be based on drugs that affect the pathomechanism of glycemic disorders and reduce cardiovascular risk, which is significantly increased in both diseases. Available options include metformin as the first-line therapy, followed by a GLP-1 RA and/or an SGLT-2 inhibitor, and finally insulin. Patient education, active monitoring, the inclusion of drugs that allow glycemic control during pasireotide treatment, and effective treatment of hypercortisolemia or GH secretion disorders may prevent cardiovascular events and improve the quality of life and survival of patients with CD or acromegaly.
